# Total Nitrogen Sources of the Three Gorges Reservoir — A Spatio-Temporal Approach

**DOI:** 10.1371/journal.pone.0141458

**Published:** 2015-10-28

**Authors:** Chunping Ren, Lijing Wang, Binghui Zheng, Andreas Holbach

**Affiliations:** 1 College of Water Sciences, Beijing Normal University, Beijing, China; 2 State Environmental Protection Key Laboratory of Drinking Water Source Protection, Chinese Research Academy of Environmental Sciences, Beijing, China; 3 Environmental Planning Institute, Sichuan Research Academy of Environmental Sciences, Chengdu, China; 4 Institute of Mineralogy and Geochemistry (IMG), Karlsruhe Institute of Technology (KIT), Karlsruhe, Germany; Wuhan Botanical Garden,CAS, CHINA

## Abstract

Understanding the spatial and temporal variation of nutrient concentrations, loads, and their distribution from upstream tributaries is important for the management of large lakes and reservoirs. The Three Gorges Dam was built on the Yangtze River in China, the world’s third longest river, and impounded the famous Three Gorges Reservoir (TGR). In this study, we analyzed total nitrogen (TN) concentrations and inflow data from 2003 till 2010 for the main upstream tributaries of the TGR that contribute about 82% of the TGR’s total inflow. We used time series analysis for seasonal decomposition of TN concentrations and used non-parametric statistical tests (Kruskal-Walli H, Mann-Whitney U) as well as base flow segmentation to analyze significant spatial and temporal patterns of TN pollution input into the TGR. Our results show that TN concentrations had significant spatial heterogeneity across the study area (Tuo River> Yangtze River> Wu River> Min River> Jialing River>Jinsha River). Furthermore, we derived apparent seasonal changes in three out of five upstream tributaries of the TGR rivers (Kruskal-Walli H ρ = 0.009, 0.030 and 0.029 for Tuo River, Jinsha River and Min River in sequence). TN pollution from non-point sources in the upstream tributaries accounted for 68.9% of the total TN input into the TGR. Non-point source pollution of TN revealed increasing trends for 4 out of five upstream tributaries of the TGR. Land use/cover and soil type were identified as the dominant driving factors for the spatial distribution of TN. Intensifying agriculture and increasing urbanization in the upstream catchments of the TGR were the main driving factors for non-point source pollution of TN increase from 2003 till 2010. Land use and land cover management as well as chemical fertilizer use restriction were needed to overcome the threats of increasing TN pollution.

## Introduction

Damming of rivers is one of the most dramatic anthropogenic impacts on freshwater environments [1–3]. Dam reservoirs significantly increase the hydraulic residence time of rivers [4] and change its flow velocity and patterns [5]. Thus, the impoundment of rivers causes changes in both hydrological and bio-chemical processes in the water body. In turn, the aquatic and riparian ecosystems are strongly affected. Every newly established reservoir is experiencing an individual cascade of environmental changes that often pose threats to both biosphere and human inhabitants. Eutrophication is amongst the most serious of these threats and has drawn wide international attention. [6].

Algal blooms are frequently the consequence of eutrophication and they often pose threats to humans and ecosystem health. Excessive nutrient loading is the major internal cause of algal blooms in closed and semi-closed water bodies such as lakes and reservoirs [7]. Nitrogen (N) and Phosphorous (P) are the two main nutrients that limit the growth of algae in freshwater [8–10]. Numerous studies have dealt with the budgets of N and/or P in reservoirs and lakes in the world [11–14]. Riverine transport is regarded as the principal pathway of pollutants into a reservoir. Nutrient availability and physico-chemical environmental conditions of reservoirs are strongly affected by the meteorological and hydrological seasonality of the reservoir itself and its whole catchment area, particularly its tributary inflows. Thus, the examination of reservoir nutrient budgets requires a regional-scale approach to obtain a profound understanding of nutrient origins.

The Yangtze River in China is the third largest river in the world, with a mean annual water discharge of 29,400 m^3^/s. The Three Gorges Dam (TGD) on the Yangtze River has created the large dentritic Three Gorges Reservoir (TGR) with a length of more than 600 km. Every year the TGR’s water level fluctuates between 145–175 m above sea level (a.s.l.) (~175 m a.s.l. from November to February, 150–170 m a.s.l. from March to May, ~145 m a.s.l. from June to August) [15]. Since the initial impoundment of TGR in June 2003, increasing numbers of intense algal blooms (3 in 2003, 26 in 2010) have been observed in its tributary backwaters [16]. The proportion of eutrophic monitoring sections in the 38 main tributaries of TGR (watershed area larger than 100 km^2^) increased from 16% in 2007 to 34% in 2010[17]. Both governmental and research institutions have agreed that eutrophication in the TGR tributary backwaters is a significant environmental threat that needs particular attention [18–20]. Moreover, the Yangtze River main stream of the TGR contains much higher nutrient concentrations than its tributaries and is also considered to be an important source of nutrients for the tributary backwaters [21]. Density current intrusions from the Yangtze River main stream provide nutrients and finally facilitate algal blooms in tributary backwaters. Since all the upstream tributaries (before Qingxicang) of the TGR contribute about 90% of the total inflow, they can also be considered being the ultimate source of nutrients in the TGR [22]. Therefore, the investigation of nutrient transport characteristics from the TGR’s upstream tributaries is essential to understand eutrophication and algal blooms in the TGR.

Prior research on TGR has been conducted about geochemistry, levels of nutrients and the effects of nutrient loading on aquatic ecosystems [23–28]. While most previous studies focused on tributary bays and the Yangtze River main stream within the TGR area, there is very little systematic analysis of upstream pollution characteristics. The Jinsha River, Jialing River, Min River, Tuo River and Wu River are the main upstream tributaries of the TGR contribution about 82% of the total TGR inflow (Qingxichang station).

In this study, we analyzed total nitrogen (TN) concentrations of the TGR’s upstream tributaries from 2003 till 2010. We used time series of TN concentrations in conjunction with discharge data from gauging stations in the upstream tributaries to assess the spatial and temporal distribution of TN pollution input from the tributaries into the TGR. Furthermore, we extracted the seasonal contribution of point and non-point sources to the pollution of TN.

## Materials and Methods

### 2.1 Study area

The Three Gorges Dam is located on the Yangtze River in China, the world’s third longest river (6300 km). Since 2003 it started impounding the famous, glorified, and widely criticized TGR. The total flooded area of the TGR is about 1080 km^2^ when water levels are raised to 175 m a.s.l [29]. The average annual precipitation in the Three Gorges area is about 900–1200 mm. The upper reaches of TGR can be divided into 5 sub-basins (Table 1).

**Table 1:** Classification of the upper TGR catchment area.

		Catchment area		Mean Discharge (1956–2010)		
Basin name	River length [km]	Absolute [km^2^]	Relative to upper reaches of TGR [%]	Absolute[m^3^/s]	Relative to TGR inflow [%]	Confluence with Yangtze River
Jinsha River	3144	485099	50%	4553.5	33.3	Yibin city
Min River	1279	135378	14%	2684.6	19.6	Yibin city
Tuo River	712	19613	2%	298.4	2.2	Luzhou city
Jialing River	1120	156142	16%	2071.6	15.1	Chongqing city
Wu River	1037	83035	9%	1546.8	11.3	Fulin county

### 2.2 Methods

#### 2.2.1 Sampling and TN determination

**Fig 1:**
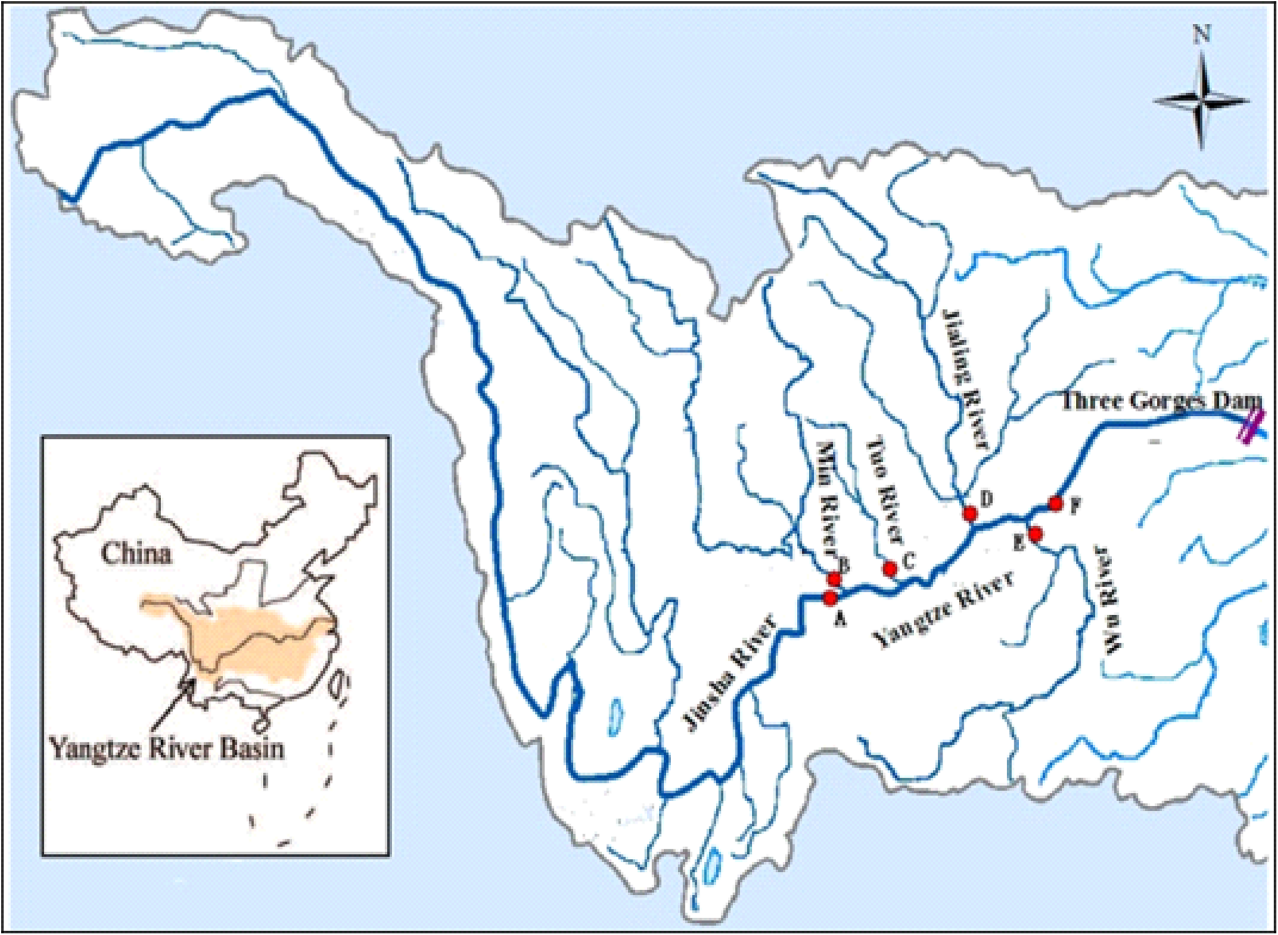
Location of study area and water quality monitoring sites.

In this study, six sampling sections were established at confluences of the Jinsha River (Shimenzi section-site A), Min River (Gaochang section- site B), Tuo River (Fushun section- site C), Jialing River (Daxiegou section-site D), and the Wu River (Wulong section- site E) with the Yangtze River main stream as well as one section at the Yangtze River main stream itself (Qingxichang section- site F). These sections were selected to represent the TN pollution status of the upstream tributaries of the TGR (Fig 1). We requested daily inflow data of each river from the corresponding hydrological gauging stations from January 2003 till December 2010. Every three months from 2003 till 2008 and monthly from 2009 till 2010, we took water samples with stainless steel containers from 0.5 m depth to analyze concentrations of TN. The study was not carried out on private land; all sampling sections are the routine water quality monitoring sections. The field studies did not involve endangered or protected species. No specific permissions were required for these locations/activities.

The sampling, preservation, transportation and analysis of the water samples were done according to the Chinese national quality standard and related national guidelines [30]. We used alkaline potassium persulfate digestion-UV spectrophotometry to determine TN concentration. In this method, water samples are oxidized by the oxidant potassium persulfate in alkaline medium at temperature of 120°C-124°C. We determine the absorptivity of nitrate at wavelength of 220nm and 275nm by UV spectrophotometric method. We used guarantee reagent Potassium nitrate (KNO_3_, GR) as reference material. Nitrogen content in both Sodium hydroxide (NaOH) and Potassium persulfate (K_2_S_2_O_8_) used in this method was less than 0.0005%. When the sample volume is 10mL, the detection limit of this method is 0.05mg/L, measured in the range of 0.20–7.00mg/L. By using this method the linear range was 0.05mg/l-7.00mg/L, the recovery was 96.3%-99.0%, the relative standard deviation was less than 2.0%.

#### 2.2.2 Statistical analysis

Time series analysis:For a water quality time series, data are usually considered as the combination of a systematic pattern and some random noise. In most cases, the random noise is considered as a white noise process with zero mean, while the systematic pattern is considered as a combination of trend, cycle and seasonality [31].It is always needed to extract the seasonal factor from the original data, when a time series with clear seasonal pattern is analyzed. Seasonal Decomposition (SD) is one of the most familiar methods to decompose the systematic pattern, which is an implementation of the Census Method I, otherwise known as the ratio-to-moving-average method [32]. Thus, this study will select SD command in SPSS 17.0 as seasonal factor extraction method. This method decomposes time-series data *X*_*t*_ into three separate components: (1) long-term trend-cycle component (*T*_*t*_), (2) seasonal component (*S*_*t*_ *)*, and (3) noise or random error (*I*_*t*_). These components can be combined by different relationships. The simplest combination relationship is either the additive or the multiplicative i.e.,

$$X_{t}=T_{t}+S_{t}+I_{t}$$ Eq 1

$$X_{t}=T_{t}.S_{t}.I_{t}$$ Eq 2

In this study, we used a multiplicative form to compute and display moving averages, *S*_*t*_, *T*_*t*_, and *I*_*t*_ for each specified series. In order to separate these components, the seasonal decomposition algorithm first estimate long-term trends applying a large-window moving-average function to the original time-series. Then, the original time-series observations are divided by these smoothed values to obtain a combined seasonal-irregular (*S*_*t*_ × *I*_*t*_) component, from which the *S*_*t*_ is isolated as the medial average for hypothesized seasonality (3 months). Seasonally adjusted values are calculated by dividing the original time-series by the *S*_*t*_. The final *T*_*t*_ is approximated by applying a second smoothing algorithm to the seasonally adjusted values. Finally, the *I*_*t*_ is calculated by dividing the seasonally adjusted series by the final *T*_*t*_ [33].

Non-parametric tests:Non-parametric statistical tests are independent of specific data distribution types and easy to operate. They are thus commonly used by scientists. The Kruskal–Wallis (K-W) test is one of these non-parametric tests. It is used to test whether samples originate from the same statistical distribution and is directly corresponding to a one-way ANOVA.

In this study, our TN concentrations dataset did not meet the requirement of homogeneity and follow a normal distribution. We tested for significant spatial and temporal differences of TN concentrations using the K-W test. As K-W test can only get the overall difference of environmental variables. We applied Mann-Whitney U to perform pairwise comparisons. We used SPSS17.0 to perform the statistical tests described above.

Hydrograph separation method:Stream-flow is considered as the combination of base-flow and direct runoff. Base-flow (dry-weather flow) refers to the portion of stream flow which is generated from groundwater and delayed shallow subsurface flow into the stream channel [34]. The estimation of base-flow from total flow is indicative in the study of hydrology. Classical techniques for base flow segmentation have been reported in numerous publications including graphical separation, recession analysis, digital filtering and conceptual models etc. [35–38]. However, in this study we used linear segmentation which is amongst the most widely used methods to calculate base flow.

Hydrograph separation method relies on the “runoff effect” in which the base flow is associated with point sources and the stream flow is associated with the non-point sources [39–44]. Therefore, in principle, separation of the runoff hydrograph into base flow and storm flow implied separation of the point source (base flow) and non-point source loads (storm flow)[41, 45]. In North America, this method is commonly used to basins where there are no sufficient data to make the non-point source loads estimation [46]. In China, this method has been used to divide the pollution load into the point sources and non-point sources of Wei River [47], Xiaoqing River [42], Hei River[43] and the Three Gorges Reservoir. Pollutants carried by base flow can be considered as the loads from point sources and natural background (hereinafter known as point source), whereas pollutants carried by direct runoff can be considered as the loads from non-point sources (hereinafter known as non-point source)[48]. The precipitation seasonality of our study area causes the base flow to occur mainly during the dry season in winter and direct runoff mainly during the wet season in summer (S1 and S2 Tables). Consequently, we considered dry season (February/March) stream-flow as the base-flow. TN loads from point and non-point sources were then estimated based on the hydrograph separation principle. The total pollutant load can then be expressed by the following equation:

$$W_{t}=\int_{O}^{t}\left[C_{p}\left(t\right)Q_{p}\left(t\right)+C_{np}\left(t\right)Q_{np}\left(t\right)\right]dt$$ Eq 3

Where *W*_*t*_ represents the total pollutants load, *t* is the time, *C*_*p*_(*t*) and *C*_*np*_(*t*) are the pollutant concentrations of point sources respectively non-point sources at time *t*, *Q*_*p*_(*t*) and *Q*_*np*_(*t*) are the base flow respectively direct runoff at time *t*.

In reality, Eq 3 always needs to be discretized to certain time steps due to the lack of continuous observation data. This can be expressed by the following equation:

$${\text{W}}_{\text{t}}=\sum_{\text{i}=1}^{\text{n}}C_{pi}Q_{Pi}\Delta \text{t}+\sum_{\text{i}=1}^{\text{n}}C_{npi}Q_{nPi}\Delta \text{t}=\sum_{\text{i}=1}^{\text{n}}C_{i}Q_{i}\Delta \text{t}$$ Eq 4

Where *C*_*i*_ is the linear interpolation value between two monitoring TN concentration,*Q*_*i*_ is the daily inflow, *C*_*pi*_ and *C*_*npi*_ are the linear interpolation values between two monitored TN concentration of point source respectively non-point sources, *Q*_*Pi*_ and *Q*_*nPi*_ are the daily base flow respectively direct runoff get by linear segmentation. In this study Δt is one day, and the TN concentration was monitored at one-month intervals.

## Results

### 3.1 Seasonal hydrology of the TGR inflow and its main tributaries

**Fig 2:**
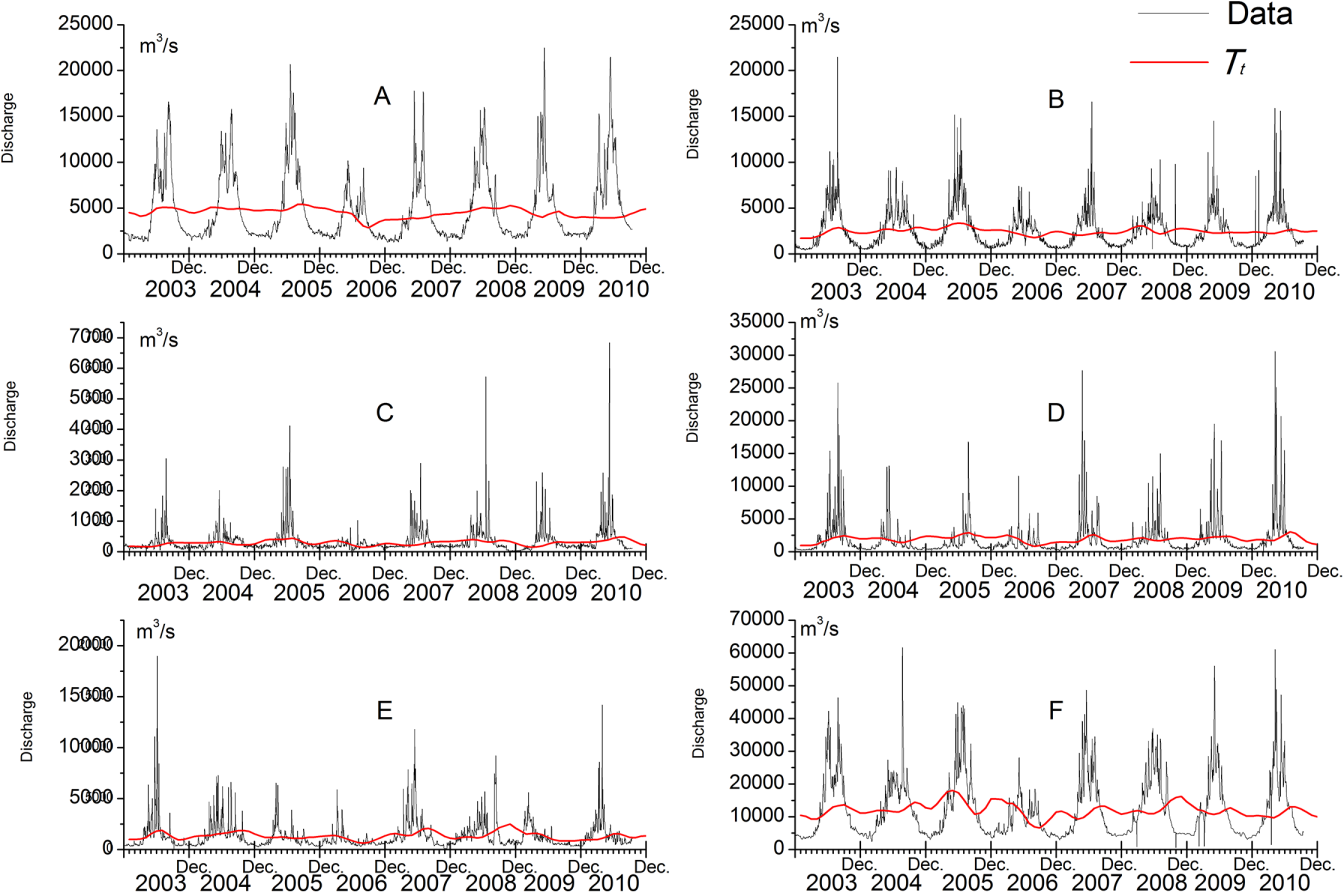
Monthly discharges (m^3^/s) variation from January in 2003 to December in 2010. A-Jinsha River, B-Min River, C-Tuo River, D-Jialing River, E-Wu River, F-Yangtze River.

The annual average (1950–2010) total inflow of TGR (site F) 393.8×10^9^ m^3^/a, accounted for 91% of the total TGR outflow (431.5×10^9^ m^3^/a). The five main tributaries that we investigated in this study contributed 357.3×10^9^m^3^/a, respectively 90% of this total TGR inflow (Jinsha River 143.6×10^9^, Min River 84.7×10^9^, Tuo River 14.9×10^9^, Jialing River 65.3×10^9^, Wu River 48.8×10^9^m^3^/a). From 2003 to 2010, the average annual inflow of the TGR was uniformly distributed between around13600 m^3^/s, only in the extreme drought year 2006 the average inflow of the TGR was exceptionally low with only 8820 m^3^/s. The subtropical monsoon climate strongly affects the TGR catchment area and causes 70% of the total runoff to occur in the rainy season between June and October (Fig 2).The highest average monthly discharge of 33300 m^3^/s occurred in July 2007 and was almost 10 times higher than the lowest discharge of 3400 m^3^/s in February 2003. Based on SD of monthly average TGR inflows, we did not find a significant long-term trend-cycle component *T*_*t*_ (11999±2126m^3^/s) for the TGR inflow (Fig 2).

All five main tributaries of the TGR did also show strong discharge seasonality (Fig 2). The highest monthly average discharges were 10 (Jinsha River) to 51 (Jialing River) times higher than the lowest ones from January 2003 till December 2010. SD of all five main tributaries did not reveal significant long-term trend cycle components *T*_*t*_ for their discharge (Jinsha River: 495±530.2 m^3^/s; Min River: 2460±336.4 m^3^/s; Tuo River: 290±78.8 m^3^/s; Jialing River: 1916±468.9 m^3^/s; Wu River: 1332±362 m^3^/s).

### 3.2 Spatial and temporal variability of TN concentration

**Fig 3:**
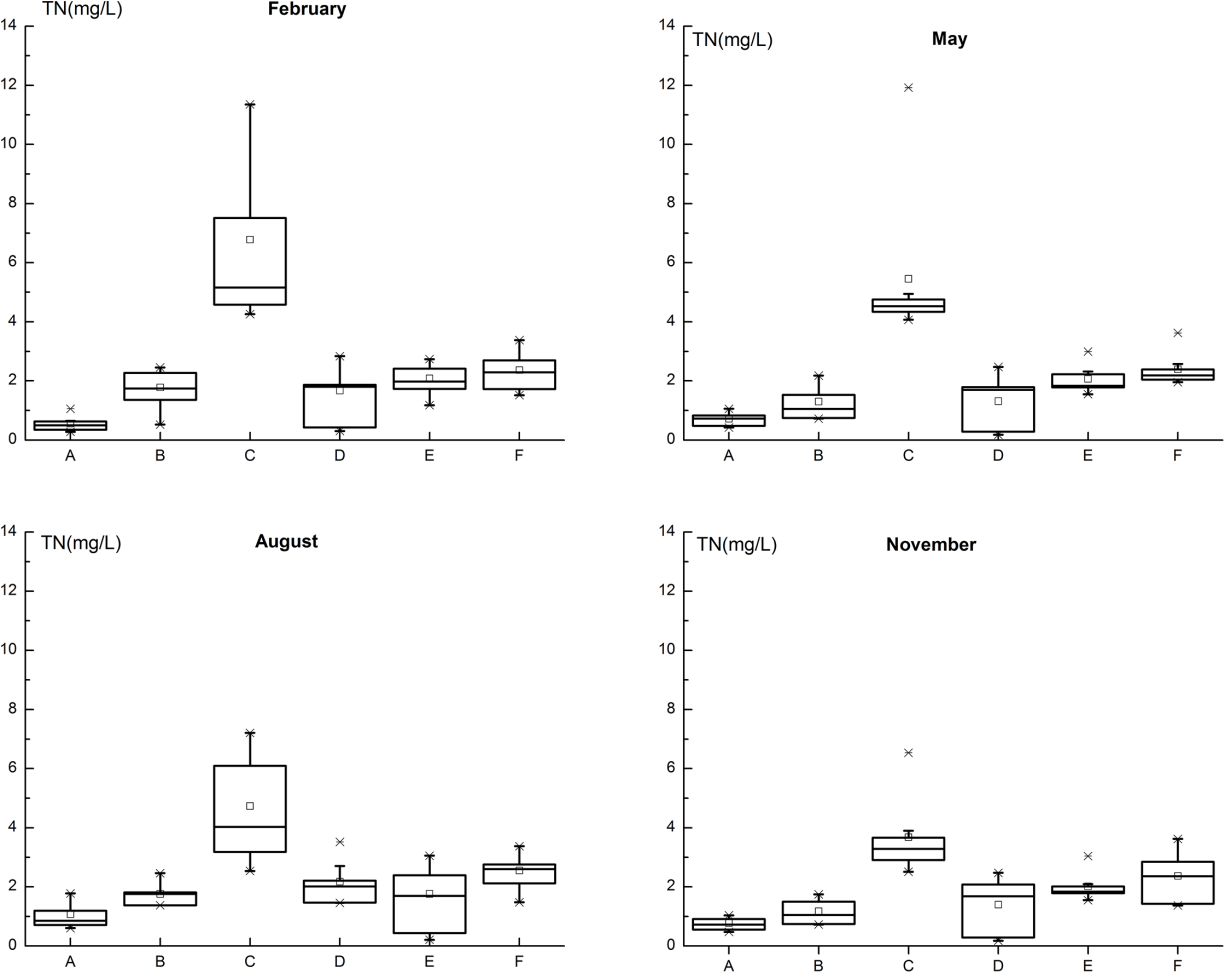
Spatial analysis of TN concentration in upstream rivers of TGR. A-Jinsha River, B-Min River, C-Tuo River, D-Jialing River, E-Wu River, F-Yangtze River.

**Fig 4:**
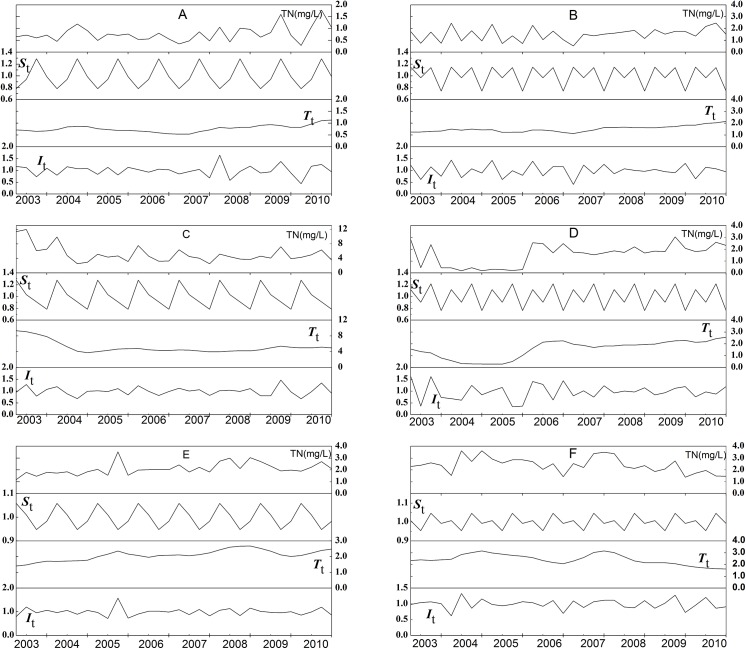
Seasonal decomposition of TN concentration in upstream tributaries of the Three Gorges Reservoir. The observed time series are decomposed in their long-term trend (*T*_*t*_), seasonal (St), and random noise (It) components. A-Jinsha River, B-Min River, C-Tuo River, D-Jialing River, E-Wu River, F-Yangtze River.

The seasonal (2003–2008) and monthly (2009–2010) monitored TN concentration at the TGR inflow section (site F) ranged from 1.37 mg/L to 3.62 mg/L. We did not identify significant seasonal TN concentration differences (ρ = 0.807>0.05) of the TGR inflow (Site F) by the Kruskal-Walli H-test (Fig 3). We could also not detect significant long-term trend cycle components *T*_*t*_ of TN concentrations at the TGR inflow (Site F) for the years 2003–2007 by SD (recycle = 4 months) (Fig 4). However, from 2008 till 2010 the SD (recycle = 4 months) did show a significant decline of *T*_*t*_ from 2.53 down to 1.67 (Fig 4).

We observed obvious spatial differences of the TN concentrations between the five investigated main tributaries (Fig 3). We found the lowest mean and median TN concentrations in the Jinsha River (0.72mg/L, 0.78mg/L) and the highest ones in the Tuo River (4.55mg/L,5.16mg/L). Inter-annual changes of TN concentrations were also obviously present for the five main tributaries (Fig 4). The Kruskal-Walli H-test revealed that these differences were significantly due to seasonality for the Tuo River (ρ = 0.009<0.05), Jinsha River (ρ = 0.030<0.05), and the Min River (ρ = 0.029<0.05). In contrast, no significant seasonal differences of TN concentrations appeared in the Jialing River (ρ = 0.667>0.05) and the Wu River (ρ = 0.926>0.05). The Mann-Whitney U-test showed that these significant differences of seasonal groups appeared between February and November for the Tuo River (ρ = 0.003<0.05) and the Min River (ρ = 0.038<0.05) and between February and August for the Jinsha River (ρ = 0.007<0.05).

### 3.3 Nutrient transport

#### 3.3.1 Temporal trends of TN transport

**Fig 5:**
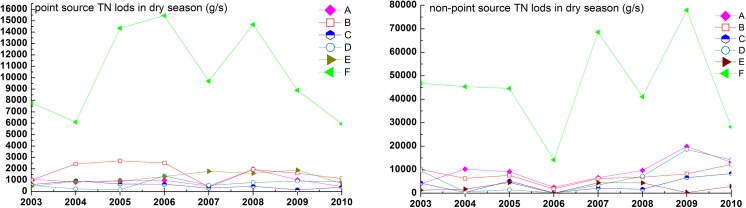
Trend analysis of TN load from point (dry season) and non-point source (wet season) in upstream rivers of TGR from 2003 to 2010. A-Jinsha River, B-Min River, C-Tuo River, D-Jialing River, E-Wu River, F-Yangtze River.

Limited by monitoring data, annual TN loads from 2003 to 2008 in upper reaches of the TGR could not be reasonably calculated in this study. However, we were able to compare the datasets of the typical dry seasons (February) with those of the wet seasons (August) to analyze the long-term trend of point and non-point source TN loads. The average load of TN in August was 3 to 11times higher than that in February. According to base flow separation principle described in the methods section, we considered the TN load in February as point source pollution carried by base flow runoff, and the TN load difference between August and February as pollution from nonpoint sources carried by direct runoff. Thus, we derived TN pollution loads for the TGR inflow (site F) around 10366±3910.6 g/s from point-sources. During the wet season (August), however, we estimated the TN pollution loads from non-point sources to be as high as 56214.4±19429.5 g/s. The inter-annual variation of TN loads was great, but we discovered no significant trend (Fig 5). Point sources in the investigated 5 main tributaries delivered around 5120g/s TN in the years 2003 to 2008 (Jinsha River 981.1±466 g/s, Min River 1720.8±820.9 g/s, Tuo River 529.9±244.8g/s, Jialing River 684.2±394.5g/s, Wu River 1203.8±529.5 g/s).The load of TN from point sources revealed an obvious decreasing trend in the Tuo River and increasing trend in the Wu River. The other three tributaries did not show clear trends. The total TN load from the 5 main tributaries in the rainy season (August) was around 35000 g/s (Jinsha River 10407.9±5474.7g/s, Min River 9159.7±2747.6g/s, Tuo River 4139.4±2884.1g/s, Jialing River 7593.4±6944.3g/s, Wu River 3669.1±1981.2 g/s). In the rainy season, the contribution of TN pollution from non-point sources was thus around 2.4 times higher than that from point sources. TN Load from nonpoint source in all tributaries but Wu River had an ascend trend (Fig 5).

#### 3.3.2 Quantification of TN annual loads from point and non-point sources

We estimated the total annual load of TN from point and non-point sources for the years 2009 and 2010 based on our monthly dataset using Eq 4 (Tables 2 and 3). The mean TN load of the TGR inflow (site F) from 2009 and 2010 was 83.3×10^4^ t/y. The total TN load of the 5 main tributaries accounted for 69.8% (Jinsha River 17.9%, Min Rive 15.9%, Tuo River 6.9%, Jialing River 18.6%, Wu River 10.6%) of this TN load that enters the TGR from its upstream basin. The TN load contribution rate of the 5 main tributaries (sites A-E) to the Yangtze River main stream (site F) was thus smaller than its runoff contribution of 90% (Section 3.1).

**Table 2:** Base flow segmentation of the upstream rivers of Three Gorges reservoir.

	2009				2010			
	Base flow(m3/s)	Base runoff (10^9^m3)	Total runoff (10^9^m3)	Surface runoff (10^9^m3)	Base flow (m^3^/s)	Base runoff (10^9^m3)	Total runoff (10^9^m3)	Surface runoff (10^9^m3)
Yangtze River	4746.7	149.69	364.29	214.60	4033.30	127.20	375.91	248.71
Jinsha River	1780.0	56.13	138.52	82.39	1543.30	48.67	132.06	83.39
Min River	889.0	28.04	71.48	43.45	835.70	26.35	79.57	53.21
Tuo River	47.0	1.48	9.05	7.57	87.90	2.77	13.27	10.49
Jialing River	543.3	17.14	66.78	49.64	500.30	15.78	75.61	59.83
Wu River	539.0	17.00	36.06	19.07	410.70	12.95	41.37	28.42

**Table 3:** The TN loads from upstream rivers into Three Gorges reservoir.

	TN load in 2009					TN load in 2010				
	Point source		Non-point source		Total load [10^4^ tons	Point source		Non-point source		Total load [10^4^ tons
	Absolute [10^4^ tons]	Relative to total [%]	Absolute [10^4^ tons]	Relative to total [%]		Absolute [10^4^ tons]	Relative to total [%]	Absolute [10^4^ tons]	Relative to total [%]	
Yangtze River	27.98	33.2	56.2	66.7	84.2	23.74	28.8	58.6	71.2	82.34
Jinsha River	2.43	18.3	10.9	82.0	13.3	3.3	19.9	13.29	80.1	16.59
Min River	4.48	40.7	6.5	59.1	11	4.8	31.3	10.56	68.8	15.35
Tuo River	0.62	12.7	4.3	87.8	4.9	1.14	17.8	5.28	82.2	6.42
Jialing River	3.14	21.1	11.8	79.2	14.9	2.91	18.1	13.14	81.9	16.05
Wu River	3.41	45.5	4.1	54.7	7.5	2.53	25.0	7.59	75.0	10.12

Compared to the base flow, direct runoff accounted for 62.6% of the total runoff at the inflow of the TGR (site F). In years of 2009 and 2010, TN load from non-point sources at the TGR inflow(site F) accounted for 68.9% of the total TN load. In the five main tributaries the contribution of TN loads from non-point sources ranged between 55% for the Wu River in 2009 and 88% for the Tuo River in 2009 and was 75% in total for both years and all 5 main tributaries. The pollution from non-point sources was thus the main source of TN in both the 5 main upstream tributaries and the inflow of the TGR

**Fig 6:**
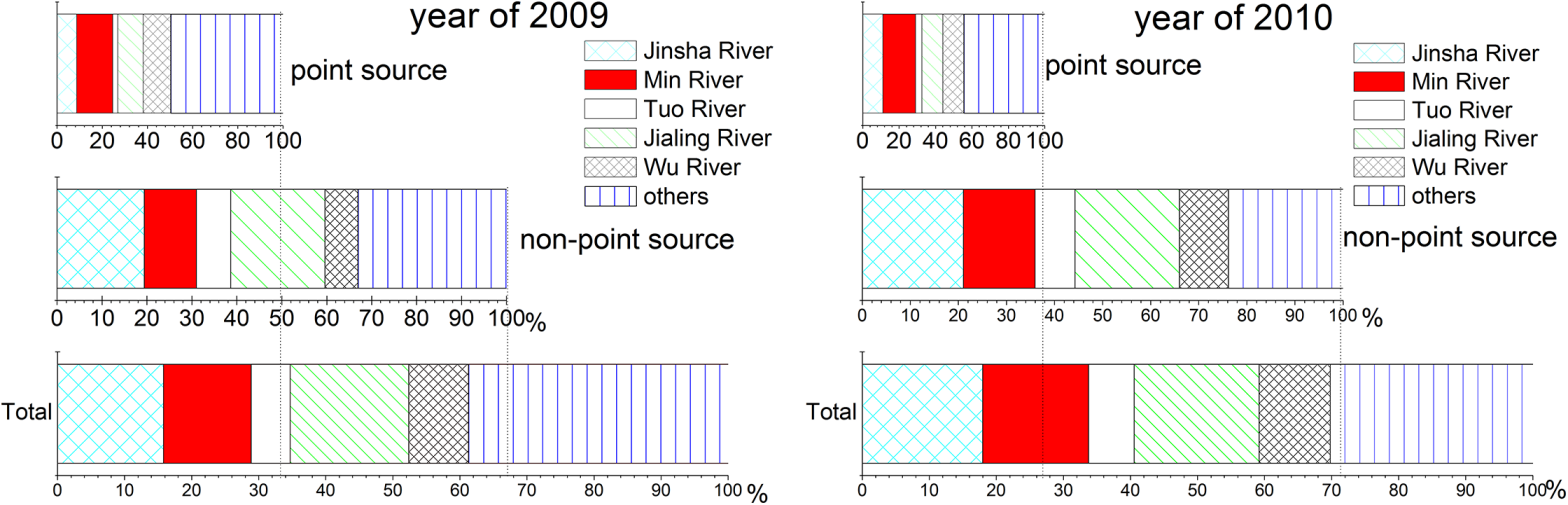
Point source and non-point source TN load contribution among the upstream rivers into TGR in 2009 and 2010.

TN pollution from point sources in the 5 main tributaries was 14.38×10^4^ t/y, which accounted for 56% of total input TN load from point sources at the inflow of the TGR (site F). TN pollution from non-point sources in the 5 main tributaries, however, was 43.72×10^4^ t/y, which accounted for 76% of the total input TN from non-point sources at the inflow of the TGR (site F). Thus, the contribution of non-point source TN by direct runoff from the 5 main tributaries is much higher than that from point sources (Fig 6).

### 3.4 Area yield of TN

Per unit area yield of nutrient could reflect the pollution intensity in the catchment. The areal TN yield of the upstream rivers of TGR was estimated based on TN concentrations, river discharge, and drainage area. Table 4 shows the areal nutrient yield of the upstream rivers of TGR. The highest non-point source areal TN yield (Tuo River, 2437.2 kg.km^-2^.yr^-1^) was 9.8 times higher than the lowest one (Jinsha River, 249.0 kg.km^-2^.yr^-1^). Both point source and non-point source areal TN yield in Tuo River basin was the highest in the study area. In contrast, both point source and non-point source areal TN yield was lowest in the Jinsha River basin.

**Table 4:** Per unit area yield of nutrient among the upstream rivers into TGR.

Basin name	River length [km]	Catchment area		pollution intensity[kg.km^-2^.yr^-1^]			
		Absolute [km^2^]	Relative to upper reaches of TGR [%]	point sources		non-point sources	
				**2009**	**2010**	**2009**	**2010**
Jinsha River	3144	485099	50%	50.1	68.0	224.7	274.0
Min River	1279	135378	14%	330.9	354.6	480.1	780.0
Tuo River	712	19613	2%	316.1	581.2	2192.4	2692.1
Jialing River	1120	156142	16%	201.1	186.4	755.7	841.5
Wu River	1037	83035	9%	410.7	304.7	493.8	914.1

## Discussion

### 4.1 Driving factors of temporal TN load variation

The TN pollution in rivers is influenced by various sources. Anthropogenic point sources include urban and industrial discharges. Non-point sources are affected by land use and land cover changes, soil types, regional topography, hydrogeological condition environmental management, etc. [36, 49–50]. Previous research reported that there were obvious positive relationship between TN load and the runoff discharge in the upstream rivers of TGR [51–52]. In this study, we found weak positive relationships between TN loads and TN concentrations (Pearson r = 0.010–0.698, p<0.01), and strong positive relationships between TN load and runoff in the five main upstream tributaries of the TGR(Pearson r = 0.795–0.930, p<0.01) (Table 5). Thus, discharge appears to be the dominant impact factor on TN load in the upstream tributaries.

**Table 5:** Pearson correlation analysis of TN load, TN concentration and discharge.

	Jinsha River	Min River	Tuo River	Jialing River	Wu River	Yangtze River
	TN load	TN load	TN load	TN load	TN load	TN load
TN concentration	0.698**	0.202*	0.010	0.421**	0.387**	0.442**
Discharge	0.896**	0.943**	0.929**	0.795**	0.903**	0.930**

*. Significant correlation at the 0.05 level (bilateral)

**. Significant correlation at the 0.01 level (bilateral)

We found increasing TN pollution from non-point sources for four (Jinsha River, Min River, Tuo River, Jialing River) out of five studied tributaries from 2003 till 2010.Especially in the rainy season (August) TN loads from non-point sources increased by 22.0%-22.4% in these rivers. Consequently, we identified direct runoff to be one of the main sources of TN pollution in the investigated rivers. Land use and land cover changes are likely to affect direct runoff and thus TN transport into rivers. Spatio-temporal changes of land use and land cover in the upstream river basins of the TGR were studied from Landsat Thematic Mapper (TM) images of 2000 and 2010 (Data available from the U.S. Geological Survey). Interestingly, the areas of forestland, grassland, and wetland in the those four river basins with increasing TN load pollution from non-point sources have increased 0.3%-3.4% from 2000 to 2010, whilst agricultural areas have decreased 0.3%-2.6% (Table 6). However, it impervious surfaces, caused growing urban areas, have increased by 27%-59% from 2000 till 2010. Furthermore, the use of chemical fertilizers has been drastically increased (19% in Chongqing municipality[53–54]; 28.2% in Sichuan province [55–56]) to feed the rapidly growing non-agriculture population of the area (46.8% in Chongqing municipality[53–54]; 31.2% in Sichuan province[55–56]). The use efficiency of fertilizers in China is generally very low, e.g. only 0.25 in the Taihu basin [57], whilst TN loss rates from cropland are serious, e.g. 3.36% in the Yangtze River delta area [58].Consequently, the negative effects of intensifying agriculture and increasing urbanization in the upstream catchments of the TGR seem to outrun the positive effects of selective land use and land cover measures like reforestation. It appears necessary to further increase governmental efforts of land use and land cover management as well as to limit and improve the use and efficiency of chemical fertilizers overcome the threats of increasing TN pollution.

**Table 6:** Dominating soil type and land use among the upstream rivers into TGR.

	TN area yield [kg/(km^2^.yr)]		Dominating soil type^a^		Land use^b^ [%]				Population density^c^[person/km^2^]
	Point sources	Non-point sources	Type	Cover [%]	Agriculture		Forest/Grassland		
					2000	2010	2000	2010	
Tuo River	448.7	2437.2	Primarosol	50.9	75.8	73.2	19.7	21.0	702
Jialing River	193.8	798.3	Primarosol	41.1	38.1	36.6	58.8	59.9	293
Wu River	357.7	705.2	Ferralosols	34.7	34.1	30.1	62.4	65.8	182
Min River	342.8	631.2	Alpine	38.9	13.4	12.0	76.1	77.0	152
Jinsha River	59.1	249.0	Alpine	49.9	7.1	6.7	72.4	72. 6	42

^**a**^ estimated through china’s 1:1,000,000 soil databases, data from institution of Soil Science, Chinese Academy of Sciences

^**b**^ estimated through Landsat Thematic Mapper (TM) images of 2000 and 2010, Data available from the U.S. Geological Survey

^c^ estimated through Chongqing Municipality, Sichuan, Yunnan, Guizhou Province Statistical yearbooks, 2010

### 4.2 Driving factors of spatial TN load variation

Spatial differences of TN pollution from point sources are dominated by industrial and urban discharges and corresponding pollution control measures such as waste water treatment facilities. In the Tuo River basin we found highest areal yields of point source TN in the study area (Table 4) which was11.6 times higher than the lowest one in the Jinsha River basin. The Tuo River basin includes the main industrial base of Sichuan Province with more than 1000 industrial plants located there [59]. The Tuo River basin also has the highest population density in our study area and includes the major cities Deyang, Neijiang, Zigong and Luzhou. Contrarily, the Jinsha River basin is weakly developed with a per capita GDP of 4000 RMB, only 60% of the national average level [55, 60]. There are fewer major cities located in the Jinsha River basin. Consequently, the results of our study show the serious impact of anthropogenic point sources on pollution loads of TN. Despite governmental efforts to improve waste water treatment efficiency in these regions, these efforts appear insufficient to really handle TN pollution anthropogenic point sources.

Nitrogen pollution from non-point sources is affected by both geogenic and anthropogenic factors [61–62]. Land use, land cover, population density, and soil type are important factors influencing non-point source pollution. The Tuo River basin is also characterized by the largest proportion (74%) of agricultural areas and the lowest proportion (20%) of forest and grassland in our study area (Table 6). It is the largest cotton and sugarcane production site of Sichuan Province. On the contrary, the Jinsha River basin hosts the lowest proportion (7%) of agricultural areas and the largest proportion (73%) of forest and grassland (Table 6). Non-point source TN pollution in the Jinsha River basin thus mainly derived from natural sources like soil erosion [63]. The sorted sequence of areal yield of non-point source TN loads is in accordance with the sequence of agricultural area proportion and is opposing the sequence of forest and grassland area proportion (Tables 4 and 6). Our results thus show a strong relationship of agriculture with non-point source TN pollution loads. Even though the total amount of agricultural area has decreased in recent years, the use of these areas was intensified by increasing use of chemical fertilizers. This intensification of agriculture seems to be responsible for increasing TN pollution loads from non-point sources in the upstream TGR area.

Population density is one of the most dynamic driving factors of pollution. On the one hand, increasing population density indirectly affects the extent and intensity of agricultural land use to sustain food supply for the people. On the other hand, non-point source TN pollution is directly affected by human emissions that are washed into surface waters by direct runoff. The sorted sequence of population density is also in accordance with areal yield of non-point source TN pollution loads.

The soil type is also one of the main factors that can affect the non-point source TN pollution intensity. The main soil types that occurred in our study area were Primarosols, Anthrosols, Alfisols, Alpine soils, and Ferralosol (Table 7). The Tuo River basin is mainly covered by Primarosols (51%), causing high erodibility and high nutrient loadings of eroded soil. The Jialing River basin is also characterized by large proportions of Primarosols (41%) and Alfisol (28%). This basin ranked second in the sequence of non-point source TN areal yield. In contrast, the dominant soil type in the other 3 River basins is Alpine soil or Ferralosols, which have a relatively low TN contains. Tuojiang River, Jialing River Basin is the most concentrated area of purple soil, which is a subclass of primarosols, and easy erosion. The average soil loss was about 7500 tons per km^2^ for purple soil [64]. This is accordance with areal yield of non-point source TN pollution loads. It again asks for increasing land cover management to limit soil erosion.

**Table 7:** soil type ^a^ and TN contents among the upstream rivers into TGR.

Basin name	Primarosols	Anthrosols	Alfisol	Alpine soil	Ferralosols
TN contents (%)[64]	0.048–0.449	0.083–0.313	0.014–0.326	0.083–0.792	0.034–0.205
Tuo River	50.9%	35.4%	4.0%	1.2%	7.2%
Jialing River	41.1%	10.9%	27.8%	4.3%	5.2%
Min River	11.7%	7.8%	26.5%	38.7%	7.0%
Wu River	34.7%	8.5%	10.8%		44.5%
Jinsha River	9.0%	1.3%	19.3%	49.9%	10.5%

^a^ estimated through china’s 1:1,000,000 soil database, data from institution of Soil Science, Chinese

## Conclusion

Runoff discharge in the main upstream tributaries of the TGR demonstrated obvious seasonality. For three of these tributaries, we observed significant seasonal changes of TN concentrations but these did not show consistent patterns between dry and rainy seasons. TN concentrations in four out of five investigated tributaries significantly increased from 2003 to 2010. The Tuo River was characterized by highest TN concentrations whereas the Jinsha River revealed lowest concentrations. The areal yield of non-point source TN was again highest in the Tuo River basin and lowest in the Jinsha River basin.

We identified land use and land cover, soil type, population density, and chemical fertilizer consumption as driving factors for the observed spatial and temporal variation of TN pollution. Pollution from non-point sources was the main contributor of TN pollution in the five main upstream tributaries of the TGR. Loads from nonpoint sources accounted for 80.9%, 84.6%, 80.6% to the total TN load in Jinsha River, Tuo River, and Jialing River respectively. Compared with 2003, non-point source TN loads in wet season in 2010 increased by 22.0%-22.4% in the Jinsha River, Min River, Tuo River and Jialing River basins. Control of TN pollution from non-point sources is especially necessary to reduce nutrient pollution in these basins. Further increase of permanent vegetation coverage and governmental regulations on limited use of chemical fertilizers appear to be effective ways to reduce TN pollution from non-point sources. In Wu River basin, TN pollution from point sources showed an increasing trend whilst TN pollution from non-point sources was decreasing. In this basin, point source pollution control must be of priority for the future management of the Wu River basin. Optimization/upgrading of waste water treatment for urban areas and industries as well as eco-compensation could become effective measures in the water environment management of the Wu River basin.

## Supporting Information

S1 File data quality analysis. (DOCX)

S1 Table Monthly Precipitation of Chongqing Municipal (2008–2010). (DOCX)

S2 Table Monthly Precipitation of Sichuan Province (2007–2010). (DOCX)
