# Supplementary material for: Total Nitrogen Sources of the Three Gorges Reservoir — A Spatio-Temporal Approach
Source: PLoS One. 2015 Oct 28;10(10):e0141458. doi: 10.1371/journal.pone.0141458 (PMC4624900; doi:10.1371/journal.pone.0141458)
Supplement: S1 File — (DOCX) [file pone.0141458.s001.docx]

**S1 Film. Data quality analysis**

In this study, we estimated TN fluxes from three-monthly/monthly TN concentration measurements paired with continuous discharge monitoring data. Combined with the hydrological segmentation of base flow, we were able to estimate the contributions of point sources and non-point sources to the total TN loads from the main upstream tributaries of the TGR. The results showed that TN load from non-point sources accounted for 55-82% to TN load in the TGR upstream tributary basins. Compared to similar previous studies, our results are within reasonable ranges [1-3] . Ideally, fluxes calculations should be based on daily concentration values and instantaneous discharges for most accurate measurements [4]. In reality however, research projects are restricted by staff and budget thus we needed to reduce our water quality monitoring frequency to three-monthly and monthly intervals described above. This reduction in monitoring frequency definitely affected the accuracy of our flux estimation. In the corresponding research field, effective estimations limited by scarce data have become a hotspot [5-7]. The reliability of such flux estimations with the given sampling frequency remains controversial [8].

In our study, we distinguished the TN loads from point and non-point sources based on the segmentation of base flow. Consequently, we assumed that pollution correlating with high discharge and thus direct runoff entirely derived from non-point sources. In fact, this assumption is not valid under all conditions. For example, point source pollution could accumulate by sedimentation at upper reaches during the dry season. This accumulated amount of pollutants would be washed downstream together with direct runoff during the rainy season. This effect could cause certain proportions of point source pollution to slip into the TN amount that we attributed to non-point source pollution. It is thus possible that the TN amounts that we attributed to non-point source pollution in this study might be slightly overestimated and the other way round. However, we consider this effect to take place rapidly after heavy rain events and it is unlikely that we took our samples in exactly this wash out time. Thus, new monitoring programs including high resolution spatial and temporal measurements in the upstream basins of the TGR are needed in order to improve the established source estimations of TN fluxes.

**References:**

1. Chong-ming LI. 1, HUANG Zhen-li~ 2 (1. Chongqing Institute of Environmental Science, Chongqing 400020, China; 2. Executive Office of the State Council Three Gorges Project Construction Committee, Beijing 100038, China); Study on the pollutant loads into three gorges reservoir (Ⅱ)——pollutant load predictions after impoundment [J]. Resources and Environment in the Yangtze Basin 2006;1.

2. Binghui Z, Lijing W, Bin G. Load of non-point source pollutants from upstream rivers into Three Gorges reservoir. Research of Environmental Sciences 2009;22(2):125-131.

3. Wang S, Yu L, Xu S, Cheng S. Research of non-point sources pollution loading in suzhou creek. Research on environmental science/Huanjing Kexue Yanjiu 2002;15(6):20-23.

4. Johnes PJ. Uncertainties in annual riverine phosphorus load estimation: Impact of load estimation methodology, sampling frequency, baseflow index and catchment population density. Journal of Hydrology 2007; 332(1):241-258.

5. Thomas RB. Estimating total suspended sediment yield with probability sampling. Water Resources Research 1985;21(9):1381-1388.

6. Walling DE, Webb BW. Estimating the discharge of contaminants to coastal waters by rivers: some cautionary comments. Marine Pollution Bulletin 1985;16(12):488-492.

7. Ferguson RI. River loads underestimated by rating curves. Water Resources Research 1986; 22(1):74-76.

8. Littlewood IG. Hydrological regimes, sampling strategies, and assessment of errors in mass load estimates for United Kingdom rivers. Environment International 1995; 21(2):211-220.
