# Supplementary material for: Total Nitrogen Sources of the Three Gorges Reservoir — A Spatio-Temporal Approach
Source: PLoS One. 2015 Oct 28;10(10):e0141458. doi: 10.1371/journal.pone.0141458 (PMC4624900; doi:10.1371/journal.pone.0141458)
Supplement: S1 Table — (DOCX) [file pone.0141458.s002.docx]

**S1 Table. Monthly Precipitation of Chongqing Municipal (2008-2010)**

|  | **2008**[mm] | **2009**[mm] | **2010**[mm] | **2011**[mm] |
| --- | --- | --- | --- | --- |
| **Jan.** | 16.2 | 26.8 | 20.8 | 14.6 |
| **Feb.** | 42.7 | 16.6 | 13.4 | 12.3 |
| **Mar.** | 43.8 | 45.2 | 47.4 | 50.6 |
| **Apr.** | 75.1 | 117.4 | 141.6 | 69.3 |
| **May.** | 91.7 | 93.5 | 121.9 | 120.5 |
| **Jun.** | 254.4 | 260.2 | 197.1 | 169.5 |
| **Jul.** | 55.1 | 102.1 | 185.0 | 85.7 |
| **Aug.** | 108.4 | 383.7 | 105.6 | 98.3 |
| **Sep.** | 54.1 | 49.3 | 57.2 | 97.7 |
| **Oct.** | 154.3 | 66.5 | 62.5 | 147.6 |
| **Nov.** | 59.8 | 18 | 56.7 | 98.0 |
| **Dec.** | 29.7 | 19.6 | 35.5 | 28.7 |
| **Total** | 985.3 | 1198.9 | 1044.7 | 992.8 |

Data from Chongqing statistical yearbook, 2009-2012
