# Supplementary material for: Total Nitrogen Sources of the Three Gorges Reservoir — A Spatio-Temporal Approach
Source: PLoS One. 2015 Oct 28;10(10):e0141458. doi: 10.1371/journal.pone.0141458 (PMC4624900; doi:10.1371/journal.pone.0141458)
Supplement: S2 Table — (DOCX) [file pone.0141458.s003.docx]

**S2 Table. Monthly Precipitation of Sichuan Province (2007-2010)**

|  | **2007[mm]** | **2008[mm]** | **2009[mm]** | **2010[mm]** |
| --- | --- | --- | --- | --- |
| **Jan.** | 10.7 | 10.1 | 7.8 | 2.0 |
| **Feb.** | 6.6 | 17.9 | 5.0 | 5.5 |
| **Mar.** | 13.0 | 36.9 | 19.3 | 42.7 |
| **Apr.** | 76.5 | 61.0 | 74.5 | 68.0 |
| **May.** | 94.2 | 132.0 | 80.0 | 108.7 |
| **Jun.** | 158.2 | 138.4 | 141.2 | 174.4 |
| **Jul.** | 204.3 | 142.0 | 192.3 | 204.8 |
| **Aug.** | 130.9 | 183.3 | 164.5 | 186.1 |
| **Sep.** | 123.0 | 106.9 | 116.2 | 134.4 |
| **Oct.** | 64.8 | 72.0 | 52.8 | 59.1 |
| **Nov.** | 17.4 | 17.2 | 16.9 | 23.0 |
| **Dec.** | 8.4 | 2.6 | 8.0 | 8.0 |
| **Total** | 908.1 | 920.3 | 878.5 | 1016.9 |

Data from Sichuan statistical yearbook,2008-2011
